# Spinal muscular atrophy and anorexia nervosa: a case report

**DOI:** 10.1186/s12887-023-03915-4

**Published:** 2023-03-14

**Authors:** Siu Tsin Au Yeung, Colleen Alford, Daniel You

**Affiliations:** The Children’s Hospital Westmead, Corner Hawkesbury Road and Hainsworth St, Westmead, NSW 2145 Australia

**Keywords:** Spinal muscular atrophy, Anorexia nervosa, Eating disorder

## Abstract

**Background:**

Spinal muscular atrophy (SMA) is an autosomal recessive condition affecting lower motor neurons causing progressive muscle atrophy. Anorexia nervosa (AN) is a psychiatric disorder characterised by intense fear of weight gain, restriction of energy intake, and preoccupation with body weight and shape. Low weight, gastrointestinal dysmotility, and respiratory infections are common in SMA but may mask AN. No paediatric cases of AN in SMA have been reported to date.

**Case presentation:**

A 14-year-old female with SMA2 presented with 12 months of declining body weight to a nadir of 24.8 kg (BMI 11). This was initially attributed to medical complications including pneumonia and gastroenteritis, and chronic gut dysmotility associated with SMA. Despite almost 2 years of dietetic input and nutritional supplementation due to the weight plateauing from age 11, no significant restoration or gain was achieved. The Eating Disorder Examination-Questionnaire (EDE-Q) indicated a possible eating disorder and psychiatric evaluation confirmed AN.

Initial management prioritised close medical monitoring and outpatient weight restoration on an oral meal plan. Skin fold anthropometric measurement was conducted to determine a minimum healthy weight. Individual psychological therapy and family sessions were undertaken. The patient developed major depression and a brief relapse with weight loss to 28 kg. Since then, the patient has maintained a weight of around 35 kg with stable mood.

**Conclusions:**

Low body weight, feeding issues, gastrointestinal dysmotility, and respiratory infections are common in SMA and diagnostic overshadowing can lead to delayed recognition of anorexia nervosa. Change to growth trajectory and prolonged weight loss should prompt consideration of comorbid psychiatric issues. Screening measures such as the EDE-Q and DASS may be helpful in this population. Close liaison between the neurogenetics and psychiatry teams is helpful. Skin fold anthropometry can assist in identifying a minimum healthy weight range.

## Background

Spinal muscular atrophy (SMA) is an autosomal recessive condition affecting lower motor neurons causing progressive muscle atrophy [1]. The incidence is 1 per 10,000 [2]. The disease is typically classified from spinal muscular atrophy type 1 (SMA1) to SMA4 by age of onset of muscle weakness and impaired achievement of motor milestones. SMA1 presents in the early infancy with diffuse proximal muscle weakness and atrophy. SMA4 has milder adult-onset weakness [1]. The cause is genetic abnormality in the survival motor neuron 1 (SMN1) gene and therapies targeting gene expression are now available. As a result, the prognosis of SMA is now more hopeful, particularly for those with earlier onset [1]. Supportive therapy aimed at nutrition and respiratory support and preventing complications of muscle weakness are cornerstones of treatment [1].

Patients with SMA often have low body weight, feeding and swallowing problems [3]. Optimal nutrition support and close monitoring of anthropometry is recommended [4]. The altered body composition, particularly lower lean body mass, in SMA makes interpretation of standard growth charts difficult [5].

Mental health comorbidity of paediatric patients with SMA has not been extensively studied. Evidence indicates that there is a high prevalence of anxiety and depression in school-aged children in China [6]. Qualitative research suggests that patients with SMA report their disease contributes to negative mental health including anxiety and depression [7, 8]. Anorexia nervosa (AN) is a psychiatric disorder characterised by intense fear of weight gain or becoming fat, restriction of energy intake, and preoccupation with and distorted perception of body weight and shape [9].

There have been no published case reports regarding anorexia nervosa or other eating disorders in paediatric SMA. Here we report a rare clinical case of AN in SMA and the complex interactions between both disorders that contributed to delayed diagnosis and impacted management.

## Case presentation

A 14-year-old female with SMA2 was referred for psychiatric review with progressively declining body weight over 12 months. Despite incremental weight gain in the decade prior, the patient’s weight plateaued at approximately 29 kg from age 12 (Basal Metabolic Index (BMI) 18) and fell to a nadir of 24.8 kg (BMI 11, < 3^rd^ centile) at age 14.

Due to the SMA, the patient was wheelchair-bound and dependent on full-time assistance since early childhood. She had multiple complications including bilateral lower limb contractures, scoliosis, osteoporosis, obstructive sleep apnoea requiring respiratory support, and gastroparesis. The patient was able to attend mainstream schooling with some educational support. Her 12-year-old brother also had SMA2, adding to the significant burden on the caregivers. The patient had no previous psychiatric history nor was there any family history of mental health issues.

Initially, the patient’s poor weight gain and subsequent loss was attributed to several medical complications including pneumonia and gastroenteritis, and chronic gut dysmotility associated with SMA. Over a 2-year period, this was managed with dietetic input and with minimal improvement. With increasingly severe weight loss by age 14, the patient also began to demonstrate increasing social withdrawal and decreased interest in usual activities prompting the neurology team to administer the Eating Disorder Examination-Questionnaire (EDE-Q) and the Depression Anxiety Stress Scale (DASS) which indicated possible AN. This triggered a referral to the psychiatry team. There was no previous involvement of mental health teams.

On psychiatric review, the patient was vague and guarded regarding her diet. However, with prompting around the EDE-Q responses, she acknowledged extreme restrictive eating practices, body image preoccupation/distortion, intense fear of weight gain, and calorie counting. A diagnosis of AN and comorbid depression was made. It was formulated that lifelong disability, chronic carer burden with two disabled children, the life-limiting nature of SMA, the restrictions on adolescent individuation, and subsequent loneliness contributed to the development of the patient’s depression. Together with the undiagnosed and untreated depression, chronic feeding and swallowing difficulties associated with SMA made the patient more vulnerable to developing an eating disorder. For the patient, food restriction was seen as a powerful way to exert control and independence, particularly in association with tendencies to internalise emotions, perfectionistic traits, and low self-esteem. The patient was unsurprised by the AN diagnosis and described relief at having it finally recognised because for so long the SMA had been her family’s overriding focus. The parents were extremely surprised by the diagnosis.

Initial management included close medical monitoring and outpatient weight restoration. Following principles of Family Based Treatment, the parents were initially empowered to take responsibility for ensuring the patient undertook increased oral intake. Skin fold anthropometry was used to determine a minimum healthy weight of approximately 30 kg. Body composition and nutritional assessment by anthropometry is used clinically in SMA, as opposed to standard growth charts which are not appropriate in this population. Family sessions initially prioritised helping the family to find their roles in supporting recovery, psychoeducation, externalising the eating disorder, and meal support skills, and later on emotional attunement. Individual psychological therapy focussed on skills in emotional regulation, challenging eating-disordered cognitions and behaviours, and improving insights on the function of AN as a motivation to rely on family as emotional support. The supplementary individual component intended to provide a private therapeutic space for the patient to promote the process of individuation. Such process was hypothesised to have been interrupted by SMA due to the patient’s reliance of caregivers for everyday needs, which then have contributed to the maintenance of AN. The patient developed major depression, likely worsened by the removal of her usual coping strategy of restriction, and was commenced on fluoxetine and olanzapine. Following a 9-month period of weight restoration and stability, there was a relatively brief relapse with weight loss to 28 kg requiring brief inpatient admission and further intensive outpatient care. Since this time, the patient maintained a weight of around 35 kg with stable mood and was discharged by the psychiatric service. Given the multi-dimensional nature of the formulation, it was believed that the combination of family work, individual psychotherapy, and pharmacotherapy were all vital and contributed to the final positive outcome of the patient’s AN and depression.

## Discussion and conclusions

This case report is the first to describe AN or any eating disorder in paediatric SMA. It highlights the interplay of multiple aspects of each disease which have implications for diagnosis, monitoring, and management. As a single case report, this is limited in contributing to the literature, and further case series and research into the interplay of these two conditions is needed.

Low body weight, feeding issues, gastrointestinal dysmotility, and respiratory infections are common in SMA [1] and the resultant diagnostic overshadowing in this case lead to a delayed recognition of AN. Significant change to growth chart trajectory, prolonged weight loss (or failure of gain) despite intensive dietetic input and nutritional supplementation should prompt early consideration of comorbid mental health issues. It is increasingly recognised that psychiatric illness is more common in the SMA population [6–8]. This case highlights the need for increased awareness of AN in SMA patients with depression. Screening measures such as the EDE-Q and DASS may be helpful in this population. When indicated, psychiatric follow-up and intervention should be included as standard of care for SMA patients for the prevention of severe and life-threatening psychiatric disorders such as AN and depression.

Due to the complex medical aspects and complications associated with SMA, close liaison between and joint review by the neurogenetics team and psychiatry team is helpful. The muscular atrophy and low lean muscle mass in SMA mean that standard growth charts are less helpful [10]. Instead, skin fold anthropometry may be helpful to identify a minimum healthy weight range and guide refeeding. As occurred in this case, families may be surprised by the diagnosis of AN and instead attribute weight loss to SMA-related complications. The joint medical-psychiatric approach is helpful in reinforcing the AN diagnosis and emphasising the urgency of refeeding. Furthermore, involvement by the specialist eating disorder team at the tertiary children’s hospital reinforced this and helped to reinvigorate their efforts.

The multitude of supportive cares and complications associated with SMA mandate a high carer burden and often leads to burnout [8]. As such, it was at times hard for the family to externalise the AN and they could fall into inadvertently blaming the patient for “keeping” AN. The typical, structured Family Based Treatment approach used for paediatric AN needed to be tailored and made flexible to accommodate the competing priorities of SMA care, such as daily physiotherapy. For example, the patient’s brother (who also had SMA) and one parent caring for him were usually unable to attend weekly family sessions. An important theme that emerged was pre-existing guilt held by the parents for their role in the genetic inheritance of the patient’s SMA. This was compounded further by the guilt of the patient now having another severe diagnosis of AN.

The urgency of treatment for AN is often framed by therapists as life-threatening in order to spur the family into action [11]. However, in the context of a life-limiting medical condition such as SMA, this framing had to be tempered somewhat as both conditions had their own competing medical priorities. The therapists supported the parents to shift some focus from the pragmatics of medical treatment, to also attending to the patient’s emotional needs. Furthermore, the in-depth involvement of the family in therapy and focus on promoting communication and emotional attunement within the family system was greatly appreciated by both the patient and parents.

The chronic disability and dependence associated with SMA has a significant impact on AN treatment in teenagers. The patient was unable to socialise typically or undertake the same usual teenager activities as her friends, particularly in relation to part time employment and learning to drive. This resulted in the patient feeling increasingly lonely and turning more to AN. Phase 3 of Family Based Treatment for AN usually involves an emphasis on adolescent individuation and independence [12]. This was harder to foster in the patient due to the impairments imposed by the SMA. However, gains in this domain were made with referral to an adolescent chronic illness support group and family encouragement for the patient to pursue interests beyond her physical disability, such as career-related school subjects.

Two complex and severe conditions interfaced in this patient, and it was critical that both sets of challenges were recognised and addressed in adaptable ways.

## Data Availability

Data on patient and case details are available from the author on reasonable request.

## Abbreviations

AN: Anorexia nervosa
BMI: Basal metabolic index
DASS: Depression anxiety stress scale
EDE-Q: Eating disorder examination – questionnaire
SMA: Spinal muscular atrophy
SMA 1–4: Spinal muscular atrophy types 1–4
SMN1: Survival motor neuron 1
